# SWL-1 Reverses Fluconazole Resistance in *Candida albicans* by Regulating the Glycolytic Pathway

**DOI:** 10.3389/fmicb.2020.572608

**Published:** 2020-10-16

**Authors:** Xiao-Ning Li, Lu-Mei Zhang, Yuan-Yuan Wang, Yi Zhang, Ze-Hua Jin, Jun Li, Rui-Rui Wang, Wei-Lie Xiao

**Affiliations:** ^1^ School of Chinese Materia Medica, Yunnan University of Chinese Medicine, Kunming, China; ^2^ Engineering Laboratory for National Health Theory and Product of Yunnan Province, Yunnan University of Chinese Medicine, Kunming, China; ^3^ College of Oceanology, Harbin Institute of Technology (Weihai), Weihai, China; ^4^ Key Laboratory of Medicinal Chemistry for Natural Resource, Ministry of Education and Yunnan Province, School of Chemical Science and Technology, Yunnan University, Kunming, China

**Keywords:** *Candida albicans*, SWL-1, glycolysis, resistant, natural compounds, combination

## Abstract

*Candida albicans* is a ubiquitous clinical fungal pathogen. Prolonged use of the first-line antifungal agent fluconazole (FLC) has intensified fungal resistance and limited its effectiveness for the treatment of fungal infections. The combined administration of drugs has been extensively studied and applied. SWL-1 is a lignin compound derived from the Traditional Chinese Medicine *Schisandra chinensis*. In this study, we show that SWL-1 reverses resistance to fluconazole in *C. albicans* when delivered in combination, with a sharp decrease in the IC_50_ of fluconazole from >200 to 3.74 ± 0.25 μg/ml, and also reverses the fluconazole resistance of *C. albicans in vitro*, with IC_50_ from >200 to 5.3 ± 0.3 μg/ml. Moreover, killing kinetics curves confirmed the synergistic effects of fluconazole and SWL-1. Intriguingly, when SWL-1 was administered in combination with fluconazole in a mouse model of systemic infection, the mortality of mice was markedly decreased and fungal colonization of the kidney and lung was reduced. Further mechanistic studies showed that SWL-1 significantly decreased intracellular adenosine 5’-triphosphate (ATP) levels and inhibited the function of the efflux pump responsible for fluconazole resistance of *C. albicans*. Proteomic analysis of the effects of SWL-1 on *C. albicans* showed that several enzymes were downregulated in the glycolytic pathway. We speculate that SWL-1 significantly decreased intracellular ATP levels by hindering the glycolysis, and the function of the efflux pump responsible for fluconazole resistance of *C. albicans* was inhibited, resulting in restoration of fluconazole sensitivity in FLC-resistant *C. albicans*. This study clarified the effects and mechanism of SWL-1 on *C. albicans in vitro* and *in vivo*, providing a novel approach to overcoming fungal resistance.

## Introduction

*Candida albicans* is an important opportunistic etiological commensal organism in humans. Generally, it resides asymptomatically in the digestive and vaginal mucosae in humans and causes only superficial infection in some people (Gow and Yadav, 2017). However, candidiasis infections are a common clinical infection in immunocompromized individuals (Pappas et al., 2018). Moreover, prolonged and widespread use of antifungal drugs, especially the first-line antifungal azoles drugs, has contributed to serious resistance, which has become an obstacle to the treatment of fungal infections worldwide (Alexander and Perfect, 1997; Prasad et al., 2019). However, the currently available agents are limited, and the development of new antifungal drug is slow and costly. Thus, new therapeutic drugs and antifungal methods are urgently required.

Efflux pump-mediated resistance is the most ubiquitous pathway among the variety of molecular mechanisms underlying fungal resistance (Kontoyiannis, 2000; Gong et al., 2019). The efflux protein mainly comprises two different types proteins. One is the ATP-binding cassette (ABC) superfamily, mainly consisting of the Candida drug resistance 1 (*CDR1*) and Candida drug resistance 2 (*CDR2*) genes encoding the Cdr1 and Cdr2 proteins, respectively. The other is the major facilitator superfamily (MFS) including multidrug resistance 1 (*MDR1*), which encodes the Mdr1 protein. The function of ABC proteins is dependent on ATP (Prasad et al., 2019). *C. albicans* has the capacity to decrease intracellular accumulation of harmful agents through the action of ABC family efflux pumps in an ATP-dependent manner. Glycolysis plays a critical role as a carbon and energy source in producing ATP for *C. albicans* (Sandai et al., 2016).

**Figure 1:**
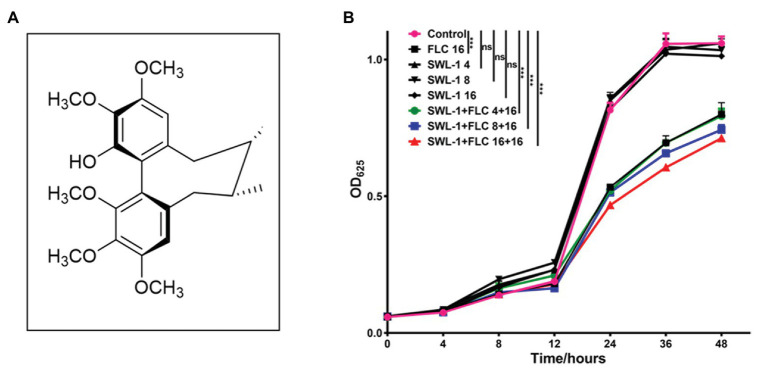
Time-course of killing of SWL-1 and FLC against FLC-resistant *C. albicans* 23^#^. **(A)** Chemical structure of SWL-1, which is derived from the Traditional Chinese Medicine *Schisandra chinensis*. **(B)** *C. albicans* 23^#^ was treated with SWL-1 or SWL-1 plus FLC at different concentrations. Each group was compared with the control group. ^***^ *p* < 0.001

We hope to find compounds from traditional medicinal plants that have antifungal or combined antifungal effects. In this study, we showed that SWL-1 is a lignin compound derived from the Traditional Chinese Medicine *Schisandra chinensis*. *Schisandra* genus species are medicinally important and commonly used in Traditional Chinese Medicine due to their diverse beneficial bioactivities. Recently, with the isolation of specific types of natural products, such as biphenyl clooctene lignans and schinortriterpenoids, secondary metabolites from this genus have attracted widespread attention from chemists and biologists (Gao et al., 2013; Liang et al., 2013; Shi et al., 2015). In this study, we aimed to discover antifungal small molecules from traditional medicinal plants by screening a small natural product library for compounds with antifungal activity using an antifungal assay. As a result, the polymethoxy biphenyl clooctene lignan (+)-Gomisin K3 (designated SWL-1; Figure 1A) isolated from the Traditional Chinese Medicine *Schisandra neglecta* (Gao et al., 2013), exerts synergistic antifungal effects when administered in combination with fluconazole (FLC) both *in vitro* and *in vivo*. Moreover, SWL-1 reversed the FLC resistance of *C. albicans* by hindering the glycolytic process to reduce the production of ATP. Thus, we showed that the glycolytic pathway was also related to drug resistance in *C. albicans*.

## Materials and Methods

### Chemical, Strains, and Culture Conditions

SWL-1 was isolated from the Traditional Chinese Medicine *S. chinensis* as described previously (Ikeya et al., 1980). SWL-1 solution (50 mg/ml) was prepared in DMSO (dimethyl sulfoxide). DCFH-DA (2',7'-dichlorofluorescin diacetate), the fluorescent probe JC-1 (5,5',6,6'-tetrachloro-1,1',3,3'-tetraethylbenzimidazolocarbocyanine iodide), Enhanced ATP Assay Kits and BCA protein assay kits were purchased from Beyotime Biotechnology (Shanghai, China). FLC was obtained from Helioeast company (Nanchang, China), and dissolved in DMSO at (50 mg/ml). BBR (berberine) was obtained from Jinke Pharmaceutical Co., Ltd. (Yunnan, China).

The standard *C. albicans* strain ATCC10231 was donated by Xue Bai of the Kunming Institute of Botany, Chinese Academy of Sciences. The clinically FLC-sensitive *C. albicans* strain 4574^#^ and FLC-resistant *C. albicans* strains 23^#^, 187^#^, 3816^#^ were donated by Professor Yu-Ye Li of the First Affiliated Hospital of Kunming Medical University of China. The FLC-sensitive *C. albicans* strain SC5314 was purchased from Yunnan Denglou Technology Co., Ltd. ATCC10231 was treated successively with FLC to obtain the FLC-resistant *C. albicans* strain (designated FLC-resistant ATCC10231). Strain 23^#^ was treated successively with SWL-1 to obtain the FLC-sensitive strain (designated SWL-1 treated 23^#^). Both strains were cultured in liquid YEPD medium (1% yeast extract, 2% peptone, 2% glucose) overnight at 30°C with constant shaking (150 rpm) before use in experiments.

### Antifungal Susceptibility Testing

The IC_50_ of SWL-1 alone and in combination with fluconazole against *C. albicans* was determined using the microdilution method (Alexander et al., 2017). Briefly, 100 μl YEPD medium was added to each well of a 96-well flat-bottomed microtiter plate before adding fluconazole and SWL-1 (final concentration 200–0.064 μg/ml in 5-fold serial dilutions). The suspension of *C. albicans* was then added to wells at a density of 10^5^ CFU/ml; the negative control contained only YEPD medium and no drugs were added in the positive control. The final volume in each well was 200 μl. Subsequently, the plates were cultured at 37°C for 24 h. Fungal growth was then determined by measurement of the optical density (OD) at 625 nm using a multi-function microplate reader. The inhibition ratio of drugs was calculated as follows:

$$\mathrm{Inhibition ratio}=100-\left(A-C\right)/\left(B-C\right)\times 100\%$$

where A, B, and C represent the OD of positive control wells, drug-containing wells, and negative control wells, respectively. The IC_50_ was calculated using GraphPad Prism 7.0 software.

To assess the effect of the interaction between SWL-1 and fluconazole, the fractional inhibitory concentration index (FICI) was calculated according to the formula FICI = FIC_FLC_ + FIC_SWL-1_. FICI ≤ 0.5 indicates a synergistic effect, 0.5 < FICI < 4.0 indicates no interaction, and FICI ≥ 4.0 indicates an antagonistic effect (Odds, 2003).

### Antifungal Kinetics Assay

To investigate the dynamic inhibitory effect of SWL-1 on *C. albicans*, time-course curves of fungal killing were plotted by measuring the OD of different groups treated with varying concentrations of SWL-1 and FLC. The overnight cultures of *C. albicans* strains were diluted with YEPD medium to 1 × 10^5^ CFU/ml and exposed to SWL-1 (4, 8, 16 μg/ml), FLC (16 μg/ml) and a combination of SWL-1 (4, 8, 16 μg/ml) with FLC (16 μg/ml); no drug was added to the control group. The cells were then incubated at 37°C with constant shaking (150 rpm) and 100-μl samples were collected from each well after 0, 4, 8, 12, 24, 36, and 48 h to measure the OD at 625 nm. Each sample was analyzed in triplicate in three independent experiments.

### Efflux Pump Assay

The effect of SWL-1 on the function of efflux pump was determined using rhodamine 6G (R6G), a fluorochrome substrate of the efflux pump (Bhattacharya et al., 2016). Briefly, approximately 1 × 10^7^ CFU/ml overnight cultures of *C. albicans* were collected after being washed three times with phosphate-buffered saline (PBS). *C. albicans* was then resuspended in PBS and cultured for 1 h in the absence of glucose at 37°C with constant shaking (150 rpm). The culture was then divided into two groups treated with and without SWL-1. These two groups were then subdivided into two more groups (four groups overall) and incubated in the presence and absence of 5% glucose to evaluate the effect of SWL-1 on the ATP-dependent and ATP-independent transporters. R6G was then added to each group at a final concentration of 10 μM and incubated for 2 h at 37°C with constant shaking (150 rpm). The absorption of R6G was terminated by placing each group on ice. Cells were pelleted and washed with ice-cold PBS to remove exogenous dye. The fungi were then resuspended in PBS before samples were removed from each group and centrifuged at 9,000 rpm for 5 min. Supernatants were collected and fluorescence was measured at an excitation wavelength 488 nm and emission wavelength 525 nm using a multi-function microplate reader.

### Measurement of Reactive Oxygen Species Production

Reactive oxygen species (ROS) generation was assayed using a DCFH-DA (2',7'-dichlorofluorescin diacetate) staining (Song et al., 2019). In brief, overnight cultures of *C. albicans* were collected, washed three times with PBS buffer, and adjusted to 1 × 10^7^ CFU/ml in PBS buffer. The fluorescent probe was added at a final concentration of 10 μg/ml and the cells were incubated at 37°C for 30 min in the dark. The cells were then collected and washed with PBS buffer before the fluorescence intensity was measured at an excitation wavelength of 488 nm and an emission wavelength of 525 nm using a multifunctional plate reader.

### Measurement of Intracellular ATP Production

Briefly, *C. albicans* were cultured overnight at 37°C and adjusted to 1 × 10^6^ CFU/ml in YEPD medium. Cells were then collected and washed with ice-cold PBS. The intracellular ATP production was then measured using ATP assay kits (Beyotime Institute of Biotechnology, Haimen, China) according to the manufacturer’s instructions (Silao et al., 2019). ATP levels in cells were calculated with reference to the standard curve, and normalized using the protein content of each sample. The results were expressed as nmol/mg protein.

### *In vivo* Antifungal Activity Evaluation Using a Murine Model of Systemic Fungal Infection

The antifungal activity of SWL-1 *in vivo* was evaluated in a murine model of systemic infection. All animals were maintained and treated in accordance with the guidelines approved by the Animal Care and Use Committee of China.

The infection model was established in male and female (1:1) BALB/C mice (aged 6–8 weeks; weighing 18–22 g). BALB/C mice were injected with cyclophosphamide *via* the intraperitoneal route (1 mg/10 g body weight) to induce immunodeficiency model before the experiment. Subsequently, the mice were randomly assigned to the following experimental groups: control (without *C. albicans*), FLC, SWL-1 (15 mg/ml), SWL-1 (30 mg/ml), SWL-1 (15 mg/ml) + FLC, SWL-1 (30 mg/ml) + FLC, BBR + FLC (positive control). BBR combined with FLC was reported to show good antifungal activity in mice systemically infected with *C. albicans* (Quan, 2006).

Mice were infected with FLC-resistant *C. albicans* suspension 10^5^ CFU/body *via* the lateral tail vein. The experimental treatments were dosed intragastrically by weight starting 2 h after model establishment. As a control, the mice in the vehicle group received only carboxymethylcellulose sodium (CMC-Na) without being infected with *C. albicans*. During successive treatments over 10 days, the general condition (activity, hair condition, weight and survival) of mice was observed and recorded. Finally, the mice were sacrificed by cervical dislocation after anesthesia, and the kidney and lung were collected and weighed before immersion in 10% buffered-neutral formalin for 24 h. Next, the fungal colonization and morphology of tissues in the different groups were evaluated following hematoxylin and eosin (H&E) and periodic acid-Schiff (PAS) staining (Kong et al., 2018; Swidergall et al., 2019).

### Determination of Mitochondrial Membrane Potential

To study the effect of SWL-1 treatment on fungal mitochondria, we measured changes in the mitochondrial membrane potential, which is a sensitive indicator of mitochondrial function and reflects the integrity of mitochondrial function (Zhang et al., 2016; Zheng et al., 2018). In brief, *C. albicans* was cultured overnight and washed twice with PBS. Cells (1 × 10^7^ CFU/ml) were then co-incubated with fluorescent probe JC-1 at a final concentration of 10 g/ml in the dark for 10 min. Subsequently, the cells were washed twice with PBS and resuspended in PBS buffer. The red fluorescence of the resuspended suspension was measured at an excitation wavelength of 550 nm and an emission wavelength of 600 nm with the full-wavelength multifunctional enzyme marker. The green fluorescence was measured at an excitation wavelength of 485 nm and an emission wavelength of 535 nm. The membrane potential was determined by calculating the ratio of red to green FI.

### Proteomics Analysis of *Candida albicans*

To understand the molecular mechanism by which SWL-1 reversed fluconazole resistance in *C. albicans*, we performed an iTRAQ-based proteomics analysis to identify differential protein expression between resistant strains and SWL-1 treated resistant strains.

### Expression of Drug Resistance Genes

To evaluate the mechanism by which SWL-1 reverses fluconazole resistance, the expression of the resistance genes *CDR1*, *CDR2*, *MDR1*, and *ERG11* was initially analyzed through qRT-PCR. Resistant strains of fungi and SWL-1 treated strains were ground into powder under liquid nitrogen, and RNA was extracted using TRIzol (Invitrogen, Carlsbad, CA, United States) according to the manufacturer’s instructions. The RNA was converted to cDNA using a reverse transcription reagent kit (Thermo Scientific) and the expression levels of resistance genes were assessed by real-time PCR (Thermo Scientific). Relative gene expression was calculated using the 2^-(∆∆Ct)^ method (Li et al., 2019). Primers for the real-time PCR analysis were designed using Primer Premier 5 and synthesized in Sangon Biotech. Primer sequences are listed in Table 1.

**Table 1:** Primers used in this study.

Genes	Primer sequences (5'-3')
*CDR1*	F: GGTGCTGCCATGTTCTTTGC
	R: AGGCATCAGCTGAAGGACGA
*CDR2*	F: AAGAGAAGCTCCATCGAGAACATTCAG
	R: CTGTCGGTTCAGCATTGGCATATAATC
*ERG11*	F: ATTGGAGACGTGATGCTGCT
	R: ATCACCACGTTCTCTTCTCAGT
*MDR1*	F: GTGCTGCTACTACTGCTTCTGGTG
	R: AACACTGATGCAATGACTGATCTGAAC

* ERG11, 14α-demethylase gene; CDR1, Candida drug resistance 1 gene; CDR2, Candida drug resistance 2 gene; MDR1, multidrug resistance 1 gene.

### Statistical Analysis

All experiments were performed in triplicate independently. All statistical analyses were performed with GraphPad Prism 7. Data were presented as the mean ± SD of triplicate experiments and differences between groups were evaluated by ANOVA. *p* < 0.05 was considered to indicate statistical significance.

## Results

### The Antifungal Effects of SWL-1 on *Candida albicans*

We determined the antifungal effects of SWL-1 on *C. albicans* strains, including the standard strain (ATCC10231 and SC5314), a clinically sensitive strain (4574^#^), clinical FLC-resistant strains (23^#^, 187^#^, and 3816^#^) and an induced FLC-resistant strain (FLC-resistant ATCC10231). As shown in Table 2, no antifungal effects on the *C. albicans* standard and clinically sensitive strains (ATCC10231, SC5314, and 4574^#^) were observed following treatment with SWL-1 alone. No interaction between SWL-1 combined with FLC was observed on the *C. albicans* standard and clinically sensitive strains, with FICIs ranging from 0.93 to 3.23. Interestingly, SWL-1 and FLC individually also had no significant inhibitory effect on the growth of FLC-resistant strains, while SWL-1 combined with FLC exhibited potent antifungal activity against FLC-resistant strains, with IC50s of 3.74–28.28 μg/ml, and FICIs of 0.13–0.38, indicating a strong synergistic effect.

**Table 2:** The antifungal activity of SWL-1 and fluconazole (FLC) alone and in combination in *Candida albicans* and the reversal of FLC-resistance in *C. albicans in vitro*.

Strains		IC_50_ (μg/ml)			FICI
		FLC	SWL-1	FLC + SWL-1	
Standard	ATCC 10231	5.06	98.92	15.78	3.23
Sensitive	SC5314	5.12 ± 1.69	94.47 ± 1.28	13.67 ± 0.41	2.81
Clinically sensitive	4574^#^	0.50 ± 0.00	91.41 ± 1.68	0.46 ± 0.13	0.93
Clinically resistant	187^#^ (FLC-resistant)	>200	90.89 ± 0.15	28.28 ± 22.17	0.38
	3816^#^ (FLC-resistant)	>200	94.51 ± 1.33	15.05 ± 3.95	0.20
Resistant	FLC-resistant ATCC 10231	>200	92.17 ± 2.01	24.45 ± 10.35	0.33
Clinically resistant	FLC-resistant 23^#^	>200	89.28	3.74	0.13
	SWL-1 treated 23^#^	5.30	—	—	—

### Kinetics of Antifungal Activity

In the antifungal kinetic assay, strong antifungal activity of SWL-1 combined with FLC was observed after treatment for 12 h (Figure 1). The results revealed that SWL-1 administered alone had no antifungal effect at 4, 8, 16 μg/ml, while FLC showed a minor effect on FLC-resistant strains 23^#^ at 16 μg/ml. In contrast, when delivered in combination with FLC at 16 μg/ml, SWL-1 exerted dose-dependent antifungal effects when administered at 4, 8, and 16 μg/ml.

After successive treatment with SWL-1, the fluconazole sensitivity of resistant strains was determined (Table 2). The IC_50_ of FLC against treatment strains reached 5.30 μg/ml, indicating that SWL-1 reverses FLC-resistance in *C. albicans*.

### *In vivo* Antifungal Activity Evaluation Using a Murine Model of Systemic Fungal Infection

**Figure 2:**
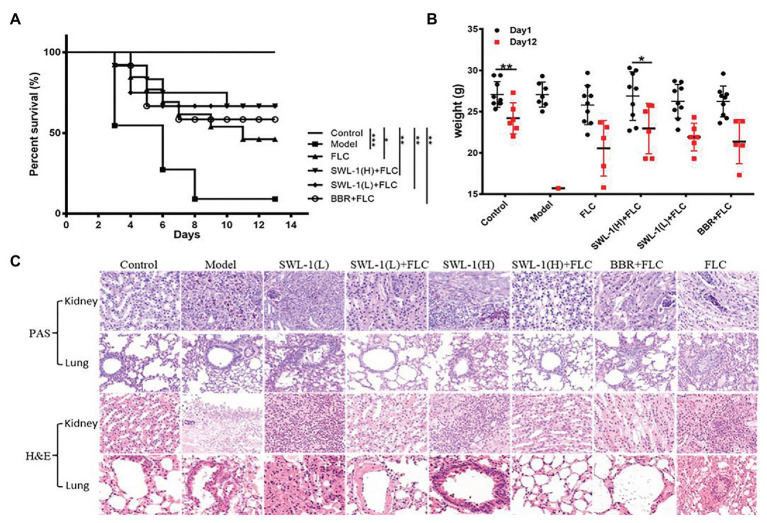
Effects of SWL-1 on fungal infection. Mice were infected with FLC-resistant *C. albicans* 23^#^ by intravenous injection (tail vein), and then treated with CMC-Na (control), FLC, SWL-1 combined with FLC, BBR combined with FLC in the model group. The condition of mice was monitored and the survival rate was calculated daily. **(A)** The weight of infected mice was recorded daily to reflect the antifungal activity of SWL-1. **(B)** Survival curves of mice infected with *C. albicans*. **(C)** Representative PAS- and H&E-stained sections of the kidney and lung from mice in the various groups 12 days after treatment. Results showed that the weight of mice in the combination therapy groups increased compared with that of mice in the model group. Each group was compared with the model group. SWL-1(H) represents the SWL-1 dose of 30 mg/ml, SWL-1(L) represents the SWL-1 dose of 15 mg/ml. ^*^p < 0.05, ^**^p < 0.01, ^***^p < 0.001.

To determine the effect of SWL-1 *in vivo*, we established a mouse model of systemic infection. After treatment with SWL-1 alone and combination with fluconazole, the survival rate and weight of mice were recorded daily. Our study showed that SWL-1 affected the survival and condition of mice. The survival rate in the model group infected with *C. albicans* was very low at less than 10%. In the SWL-1 + FLC group, the survival rate was significantly increased to more than 65% compared with that in the model group and the group treated with FLC alone (Figure 2A), indicating that SWL-1 combined with FLC increases the survival of *C. albicans*-infected mice *in vivo*.

The weight of mice reflects the general health of the mouse. As shown in the Figure 2B, there was no difference in weight of the mice in the control group between first and last day of the experimental period. The weight of the mice in the model group decreased sharply after infection with FLC-resistant strains. In contrast, the weight changes in the groups treated with low and high-dose SWL-1 combined with FLC were much slower compared with the changes observed in the FLC group and the BBR + FLC positive control group.

To better clarify effects of SWL-1 *in vivo*, we conducted histopathological and morphological studies of the kidney and lung of model mice. Paraffin-embedded sections of kidney and lung were stained with H&E and PAS. As shown in Figure 2C, there were high levels of fungal colonization in the kidney of the model group, with *C. albicans* and hyphae visible in the sections. In addition, numerous fungal cells were detected in the kidneys of mice treated with SWL-1 or FLC alone. Pathological bleeding within and from the renal tubule was observed in all the groups treated with FLC or SWL-1 alone and in the infection only group. In contrast, the kidneys of mice treated with SWL-1 + FLC or BBR + FLC (positive control) were significantly protected against the effects of fungal infection and exhibited normal morphology. Moreover, the kidneys of the model infection group and the groups treated with SWL-1 or FLC alone exhibited marked infiltration by inflammatory cells. These results demonstrated that combination therapy alleviated fungal infection. Neither hyphae nor yeast were found in the lung tissues of any of the groups. However, marked changes were observed in the morphology of the lung tissues in the model group and the groups treated with SWL-1 or FLC alone. As shown in Figure 2C, alveolar septa were thickened, with increased numbers of inflammatory cells. However, lung tissues in the SWL-1 + FLC and BBR + FLC groups were normal compared with those in the control group. Overall, these findings indicated that SWL-1 combined with FLC resulted in significant improvements in tissue pathology and that SWL-1 and FLC exert synergistic effects in the treatment of fungal infections *in vivo*.

### Expression of Drug Resistance Genes

**Figure 3:**
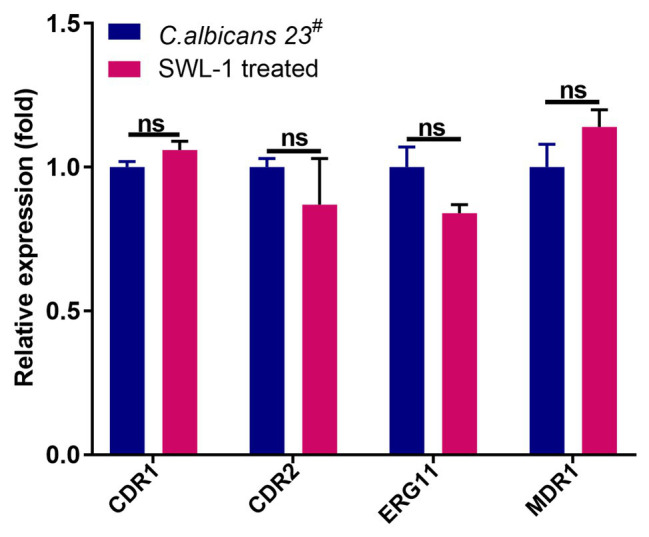
The effect of SWL-1 on resistance-related gene expression in *C. albicans*. Expression of four genes (*CDR1*, *CDR2*, *ERG11*, and *MDR1*) was detected by RT-PCR. ns, not significant.

The expression levels of FLC-resistance genes are shown in Figure 3. There were no differences in the expression levels of FLC-resistance genes between the FLC-resistant and SWL-1 treated strains.

### SWL-1 Decreases Efflux Pump Function

**Figure 4:**
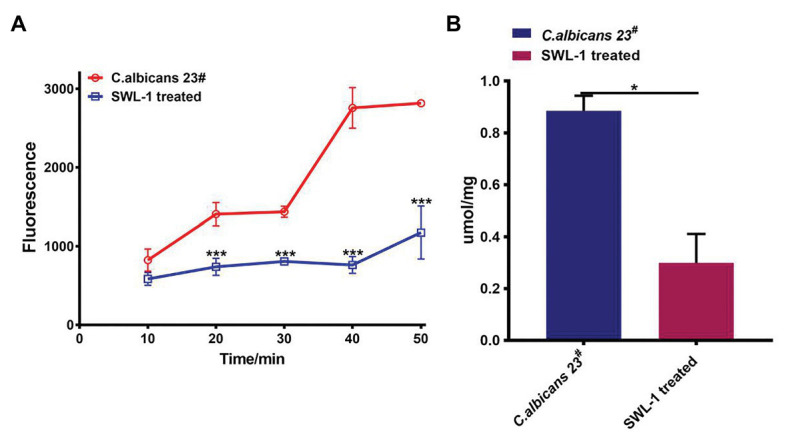
SWL-1 affected the pump activity of *C. albicans*. **(A)** Glucose-induced R6G efflux in FLC-resistant *C. albicans* 23^#^ and SWL-1-treated 23^#^. **(B)** The intracellular ATP levels in FLC-resistant *C. albicans* 23^#^ and SWL-1-treated 23^#^. ^*^p < 0.05, ^***^p < 0.001.

To investigate the effects of SWL-1 on the efflux pump of *C. albicans*, we compared the efflux activity of the FLC-resistant *C. albicans* 23^#^ and SWL-1-treated strains in R6G assays. As shown in Figure 4A, efflux of the SWL-1-treated strains was decreased significantly compared with that of the FLC-resistant, indicating that SWL-1 reverses fluconazole resistance by inhibiting the function of the efflux pump.

### SWL-1 Decreases the ATP Content of Cells

To determine the effects of SWL-1 treatment on mitochondrial function, we measured the intracellular ATP content using ATP assay kits. As shown in Figure 4B, the ATP level in the SWL-1-treated strains was markedly decreased compared to that in the FLC-resistant strains.

### SWL-1 Affects the Function of Mitochondria

**Figure 5:**
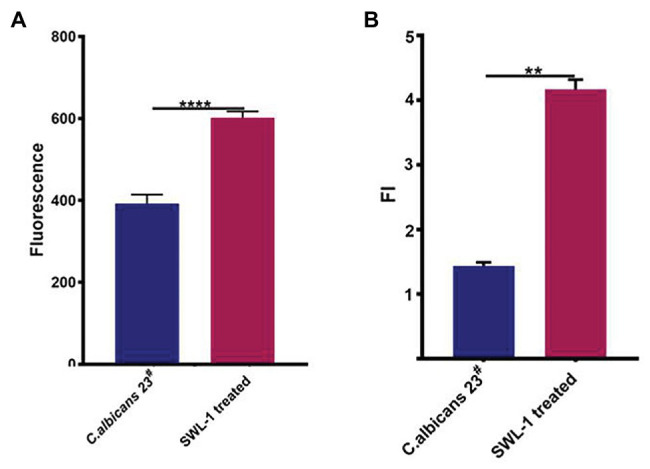
The effects of SWL-1 on the function of mitochondria. **(A)** Intracellular reactive oxygen species (ROS) production of FLC-resistant *C. albicans* 23^#^ strains and SWL-1 treatment 23^#^. **(B)** Mitochondrial membrane potential of *C. albicans* 23^#^. Data represent the mean ± SD. ^**^p < 0.01, ^****^p < 0.0001.

The production of ROS in the SWL-1 strains was obviously increased compared to that in the FLC-resistant strains 23^#^ (Figure 5A), indicating that SWL-1-treated strains increase endogenous ROS production, which may be related to mitochondrial function (Figure 5B).

### SWL-1 Changes the Expression of Glucose Metabolism-Related Proteins

**Figure 6:**
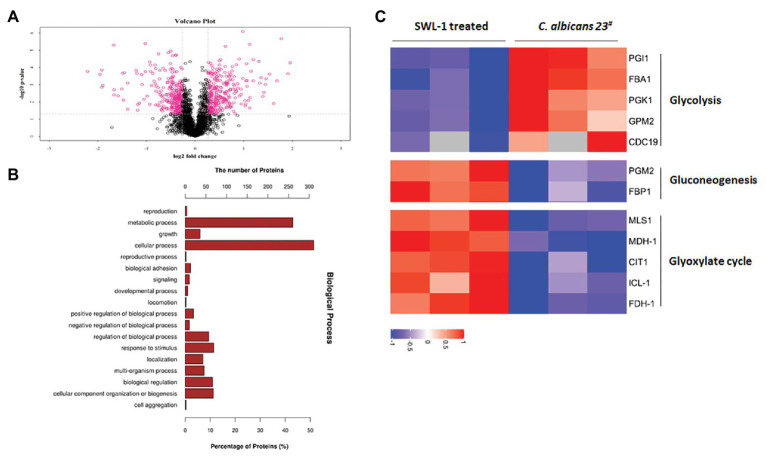
Proteins related to *C. albicans* metabolic routes identified by proteomics analysis. **(A)** Volcano map of *C. albicans* and SWL-1treated 23^#^. **(B)** Hierarchical clustering analyses of proteins that were up- or downregulated in *C. albicans* and SWL-1 treated 23^#^. **(C)** Proteins related to metabolism identified in GO analysis.

**Figure 7:**
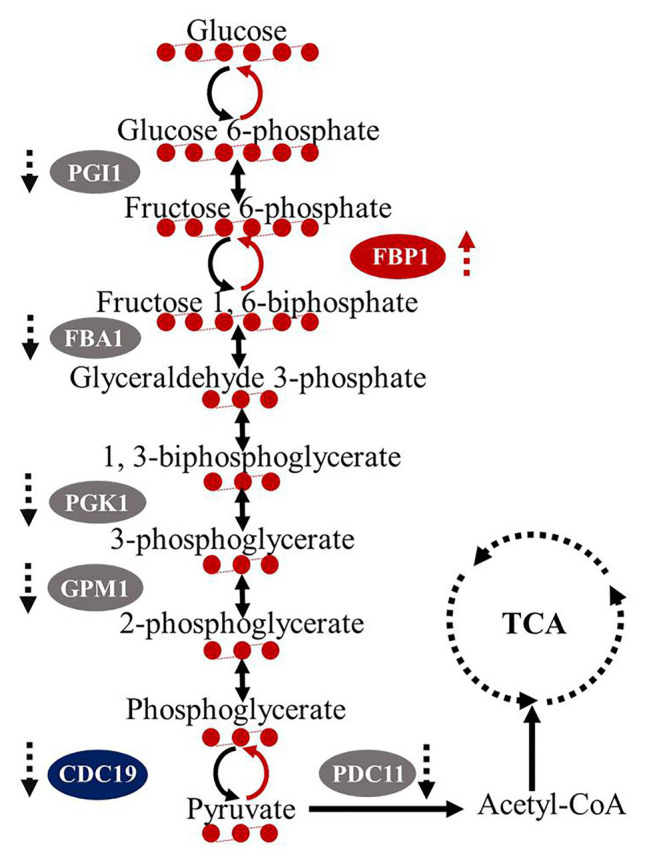
Impacts of SWL-1 treatment on glycolysis. SWL-1 inhibits some key enzymes in the glycolytic pathway, resulting in downregulation of the pathway. In contrast, some enzymes in the gluconeogenic pathway are upregulated.

Proteomics analysis of *C. albicans* 23^#^ and the SWL-1 treated strains revealed 605 differentially expressed proteins (307 upregulated and 298 downregulated) in the SWL-1treated stains compared with the *C. albicans* 23^#^ strains (Figure 6A). As shown in Figures 6B,C, hierarchical cluster analysis revealed that these differentially expressed proteins were mainly involved in metabolism, especially glucose metabolism. Phosphoglucose isomerase, aldolase, phosphoglycerate kinase, phosphoglycerate mutase, and pyruvate kinase, which are involved in the glycolysis pathway, were all downregulated, indicating repression of glycolysis (Figure 7).

## Discussion

The widespread and prolonged use of antifungals results not only in the development of tolerance toward the drug in use, but also in the development of collateral resistance to other drugs and to a variety of unrelated compounds (Prasad and Rawal, 2014). We aimed to identify compounds derived from traditional medicinal plants that exert good or synergistic effects on resistant fungi. In this study, we found that SWL-1, which is derived from *S. chinensis*, exerts synergistic antifungal effects when combined with fluconazole by inhibiting efflux pump function.

Reduced intracellular accumulation of drugs is a common mechanism of resistance in *Candida* cells, clinical azole-resistant isolates of *C. albicans* display transcriptional activation of genes encoding ABC (*CDR1*, *CDR2*) or MFS (*MDR1*) proteins. Invariably, resistant *Candida* cells, which show enhanced expression of efflux pump-encoding genes, also show a simultaneous increase in the efflux of drugs (Cannon et al., 2009; Redhu et al., 2016). Our results show that SWL-1 significantly reduced efflux pump activity without affecting the expression of the *MDR1*, *CDR1*, and *CDR2*, indicating that SWL-1 affects the efflux pump function in a gene expression-independent manner. The important characteristic feature of ABC drug transporters is that they utilize the energy generated by ATP hydrolysis to transport a variety of substrates across the plasma membrane (Prasad et al., 2006). In this study, we showed that SWL-1 inhibited the function of the ATP-dependent-efflux pump in the presence of glucose. Furthermore, the intracellular ATP content of SWL-1 treated stains was markedly decreased compared with that in *C. albicans* strains. We speculated that SWL-1 inhibits efflux pump function by inhibiting ATP synthesis.

**Figure 8:**
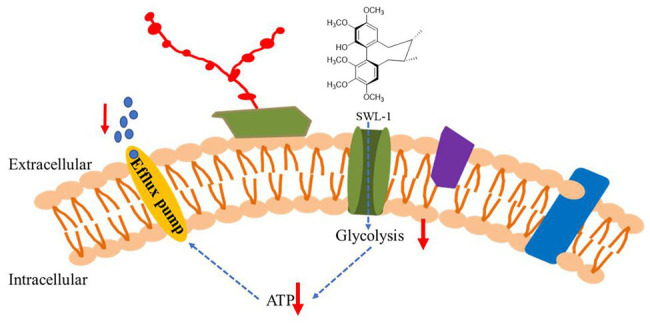
The proposed mechanisms by which SWL-1 reverses drug resistance in *C. albicans*. The glycometabolism of *C. albicans* is changed by decreasing glycolysis but increasing gluconeogenesis, resulting in a decrease in ATP levels. The efflux pump-mediated removal of FLC from cell is blocked. The resistance to FLC is reversed and the antifungal effects of FLC are restored.

Carbohydrates are the primary and preferred source of metabolic carbon for most organisms, and are used for energy and biomolecule production. In eukaryotic cells, glycolysis is a common initial glycometabolism pathway (Icard et al., 2018). Most sugars are converted to glucose 6-phosphate or fructose 6-phosphate before entering the glycolytic pathway. Glycolysis is then responsible for converting these hexose phosphates into the key metabolite pyruvate, while also producing ATP and NADH. Then, cells employ two major strategies for energy production: fermentation (without oxygen) and respiration (with oxygen). Fermentation produces lactic acid, while respiration produces additional ATP *via* the tricarboxylic acid (TCA) cycle and oxidative phosphorylation. As a central metabolic pathway, glycolysis is strictly regulated and although ATP is relatively low, glycolysis provides ATP more rapidly than the TCA cycle in mitochondria. The opportunistic human fungal pathogen *C. albicans* is a facultative aerobe and thus, metabolizes carbon sources in response to oxygen availability similar to that of a typical eukaryotic cell (Askew et al., 2009). Our proteomics data showed significant downregulation of the expression of genes related to the glycolytic pathway of drug-resistant *C. albicans* after SWL-1 treatment, thus reducing the activity of the glycolytic pathway. Since efflux pump activity is higher in resistant *C. albicans* than that in sensitive strains, ATP is more important for resistant strains to maintain the function of efflux pump. Taking these results into consideration, we propose the following hypothesis to explain the phenomenon observed in FLC-resistant strains: SWL-1 disrupts glucose metabolism, thus reducing ATP production. The energy available to the efflux pump is therefore decreased, leading to a reduction in efflux function. As a result, *C. albicans* resistance to FLC is reversed (Figure 8). Efflux pump activity is low in sensitive strains, and less ATP is used for efflux function; therefore, no similar phenomenon was observed in the sensitive strains (Table 2). The results of the present study indicate that the glycolytic pathway plays an important role in drug resistance in *C. albicans*.

Macrophage-based defense is crucial for host survival in invasive candidiasis as demonstrated by the fact that ablation of kidney macrophage numbers leads to fatal *C. albicans* infection in the mouse model. As a successful pathogen, *C. albicans* has evolved mechanisms to evade macrophage-based immunity (Lionakis et al., 2013). Glucose metabolism plays a central role in immune cell function. Following recognition of microbial ligands, macrophages upregulate glucose uptake and its anaerobic catabolism instead of relying on the TCA cycle and mitochondrial oxidative phosphorylation (OXPHOS; use of glycolysis coupled to lactic acid fermentation in normoxic conditions, i.e., aerobic glycolysis). Thus, cells obtained the required energy required to boost antimicrobial mechanisms and cytokine production. This behavior resembles the so-called Warburg effect observed in cancer cells, in which aerobic glycolysis is the main source of energy (Pearce and Pearce, 2013; Pellon et al., 2020). When macrophages are challenged with *C. albicans*, *C. albicans* rapidly consumes glucose, causing macrophage death (Tucey et al., 2018). Our *in vivo* histopathological data showed that SWL-1 + FLC treated mice had less inflammatory cell infiltration of the kidney and significant improvements in the kidney and lung lesions were observed. Thus, we speculate that SWL-1 also regulates antifungal immunity by inhibiting the glycolytic pathway.

In this study, we preliminarily elucidated the mechanism of action of SWL-1 on fluconazole-resistant *C. albicans*. More importantly, the combination of SWL-1 with fluconazole was shown to reverse *C. albicans* resistance to fluconazole *in vitro* and *in vivo*. Furthermore, proteomics analysis showed that SWL-1 hinders the glycolysis process in *C. albicans*. We speculate that SWL-1 may reverse drug resistance in *C. albicans* by reducing the activity of the glycolytic pathway. Our findings highlight new strategies for the treatment and reversal of fungal resistance and are of great significance for the treatment of *C. albicans* infection, although the underlying molecular mechanism remains to be fully elucidated.

## Data Availability Statement

The datasets presented in this study can be found in online repositories. The names of the repository/repositories and accession number(s) can be found in the article/Supplementary Material.

## Ethics Statement

The animal study was reviewed and approved by Animal Experiment Ethics Review Committee of Yunnan University of Traditional Chinese Medicine.

## Author Contributions

R-RW, Y-YW, and W-LX designed the experiments. X-NL, L-MZ, YZ, Z-HJ, and JL performed the experiments and interpreted the data. X-NL, L-MZ, and R-RW wrote the manuscript. All authors contributed to the article and approved the submitted version.

### Conflict of Interest

The authors declare that the research was conducted in the absence of any commercial or financial relationships that could be construed as a potential conflict of interest.

## Supplementary Material

The mass-spectrum data of *Candida albicans* proteomics were deposited in ProteomeXchange with the primary accession code PXD019736.
