# Supplementary material for: SWL-1 Reverses Fluconazole Resistance in Candida albicans by Regulating the Glycolytic Pathway
Source: Front Microbiol. 2020 Oct 16;11:572608. doi: 10.3389/fmicb.2020.572608 (PMC7596347; doi:10.3389/fmicb.2020.572608)
Supplement: Supplementary file 1 [file Data_Sheet_1.doc]

Supplementary Material

**Table 1** 23^#^ strain drug resistance

| Drug | Fluconazole | Voriconazole | Itraconazole | Amorolfine | 5-fluorocytosine |
| --- | --- | --- | --- | --- | --- |
| IC_50_(µg/mL) | >200 | >200 | >200 | 32.32±26.36 | 1.47±0.69 |

We have conducted drug sensitivity tests on 23^#^ . The result showed that 23^#^ is resistant to fluconazole, itraconazole and Voriconazole, and sensitive to amolofine, 5-fluorocytosine . It indicated that 23^#^ is an azole resistant strain.


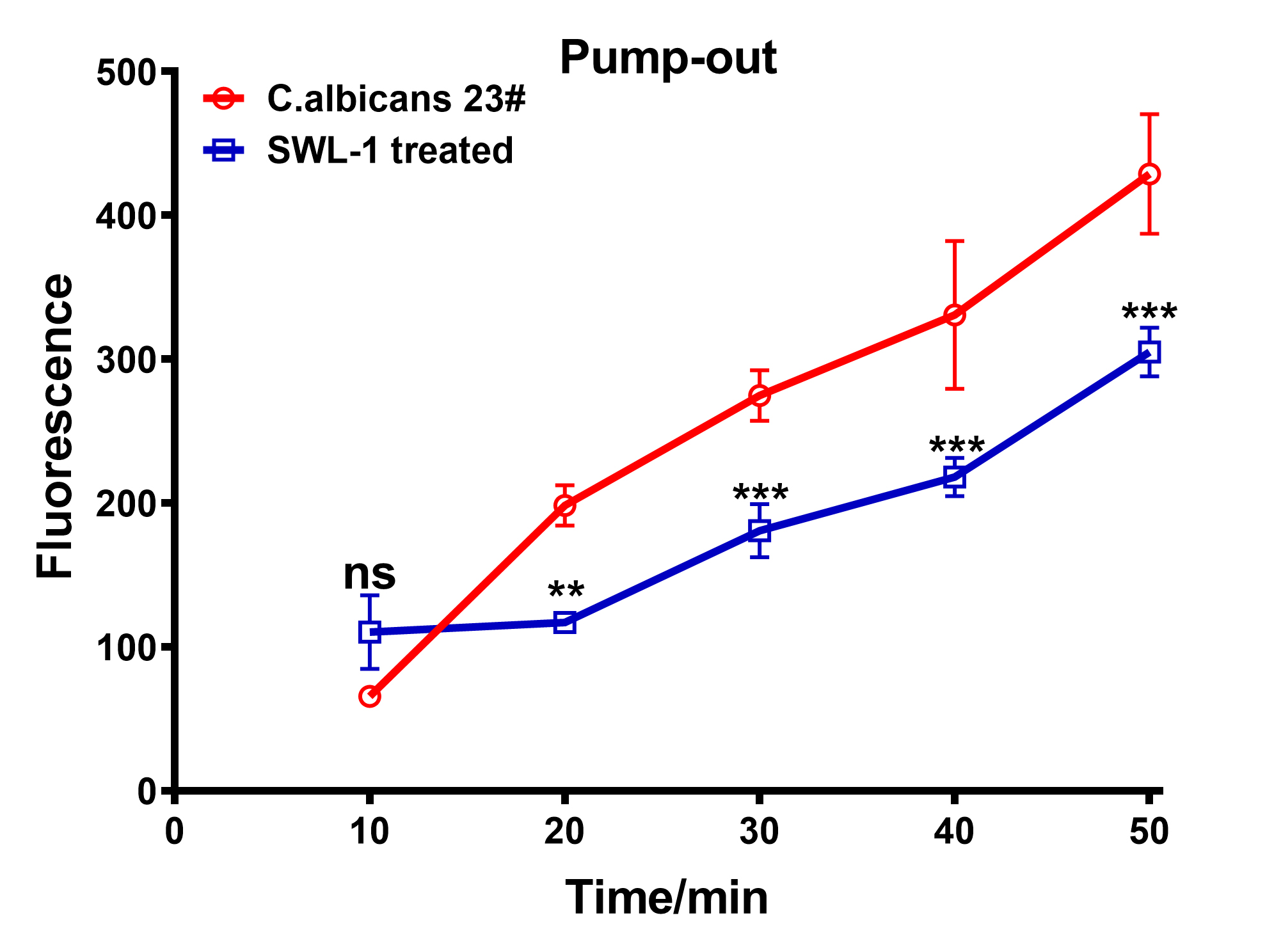


**Figure S1.** R6G efflux in FLC-resistant *C. albicans* 23^#^ and SWL-1-treated 23^#^ without glucose

The efflux pump function is expressed in drug-resistant strains. Fluconazole is pumped outside *Candida albicans* cells by efflux pump proteins, so there is not enough fluconazole to destroy the fungal cell membrane. SWL-1 inhibits the efflux pump function especially ATP-dependent efflux pump(Figure 4A), but less efficient on ATP-independent efflux pump(figure S1), so we think SWL-1 inhibits the efflux pump function is related to ATP generation. When combined with SWL-1, ATP generation was inhibited, then fluconazole is not pumped out, so it accumulates inside the fungal cells to destroy the fungal cell membrane, thereby restoring the sensitivity of drug-resistant strains. In the WT strain, the efflux pump protein is not expressed, fluconazole destroy the fungal cell membrane without the need of ATP, so SWL-1 has no visible effect on WT strain. Since ATP- dependent efflux pump function is very important in azole-resistant strains, we think a large amount of ATP was used for the activity of efflux pump in resistant strains. But ATP has other important physiological functions, SWL-1 inhibited ATP production in WT and drug-resistant strains may have other effects that need to be further investigated.
